# The past, present, and future of research on neuroinflammation-induced mild cognitive impairment: A bibliometric analysis

**DOI:** 10.3389/fnagi.2022.968444

**Published:** 2022-07-29

**Authors:** Ming Cai, Yuanqi Zhang, Si Chen, Zhan Wu, Lei Zhu

**Affiliations:** ^1^College of Rehabilitation Sciences, Shanghai University of Medicine and Health Sciences, Shanghai, China; ^2^School of Sports Sciences, Qufu Normal University, Qufu, China; ^3^The Affiliated High School of Qufu Normal University, Qufu, China

**Keywords:** mild cognitive impairment, neuroinflammation, CiteSpace, VOSviewer, microglial, astrocytes

## Abstract

**Background:**

Mild cognitive impairment (MCI) is a precursor to dementia, and neuroinflammation in the brain is thought to be one of the main pathogenic mechanisms of MCI. However, the underlying neurobiological mechanisms have not been fully explored. The purpose of this study was to establish a visual model map of the articles in the field of neuroinflammation-induced MCI over the past 11 years to reveal the research hotspots and predict the future development trends in this field, which will help to promote the research and development for MCI.

**Methods:**

The “neuroinflammation” and “mild cognitive impairment” were used as search terms, and literature about neuroinflammation-induced MCI published between 2011 and 2021 was collected from the Web of Science. CiteSpace and VOSviewer were used to create visual model maps, and assess collaboration among different authors, countries, and institutions. Finally, the current research hotspots and future research directions were analyzed by using high-frequency keywords analysis and co-cited reference burst analysis.

**Results:**

A total of 226 articles were retrieved. The number of publications in neuroinflammation-induced MCI shows an upward trend. Since 2018, the number of papers published in this field has increased significantly, with an average of more than 100 published each year. The United States had the highest literature production and the number of cited journals in this research area, and the National Institute on Aging was the most productive research institution. Brooks D.J. and Heneka M.T. had the highest number of publications and had the highest frequency of co-citations. The co-cited references revealed the evolution of the research themes, and the current studies are mainly focused on the effects of various metabolites on the control of microglial activation. “Cerebrospinal fluid,” “mouse model,” “tau,” “microglial activation,” “astrocytes,” and “TREM2” were the current high-frequency and emerging keywords.

**Conclusion:**

Research on neuroinflammation-induced MCI is burgeoning, and the close collaboration with different nations and institutions need to be further strengthened. Current research hotspots are focused on the effects of various metabolites on microglia activation. Future studies should focus on how to regulate the phenotypes of microglia and astrocyte to reduce neuroinflammation and treat MCI.

## Introduction

Mild cognitive impairment (MCI) is a risk precursor to dementia, which has a high conversion rate to dementia, especially Alzheimer’s disease (AD) (Liu et al., 2021). There is no effective treatment for AD, and timely intervention during MCI could significantly reduce the prevalence of AD. Neuroinflammation is a brain’s defense mechanism, which initially protects the brain by removing or suppressing various pathogens. However, persistent inflammation is detrimental because it inhibits neurogenesis, and it is also a significant driver of MCI onset and progression to AD (Kwon and Koh, 2020; Zhou et al., 2021).

The primary cause of neuroinflammation is the activation of microglia and astrocytes. Microglia are resident immune cells of the central nervous system (CNS) and play an essential role in tissue homeostasis. Once activated, microglia can be categorized into M1 pro-inflammatory phenotype and M2 anti-inflammatory phenotype. Although this classification may be too simplistic, it still helps to illustrate the two primary functional features of microglia (Ransohoff, 2016). In endogenous or exogenous pathological injury, activated microglia transform into M1 or M2 phenotypes. Under normal physiological conditions, M1-type microglia secrete pro-inflammatory factors that enhance cytotoxicity to promote synaptic pruning or kill foreign pathogens, and M2-type microglia promote inflammation resolution and tissue healing by sensing injury signals and secreting anti-inflammatory neuroprotective factors. This dynamic balancing process is beneficial for maintaining synaptic plasticity (Bajetto et al., 2002; Owens et al., 2014; Solé-Domènech et al., 2016). As for astrocytes, their functions are analogous to housekeeping, including forming the outer layer of the blood-brain barrier (BBB), regulating blood flow, and providing energy metabolites for neurons. Moreover, astrocytes are also important modulators of neuroinflammation in the CNS, secreting pro-inflammatory (A1 phenotype) or anti-inflammatory (A2 phenotype) cytokines depending on the stimulus. When inflammation occurs, the pro-inflammatory A1 phenotype is activated *via* the NF-κB pathway, producing pro-inflammatory factors that may contribute to pathogen and toxin clearance. But once A1 astrocytes lose their homeostatic function, they will secrete soluble toxins to kill neurons and oligodendrocytes. What toxin is responsible for this killing effect is yet to be further investigated (Huang et al., 2017). By contrast, cerebral ischemia induces the A2 phenotype through the signal transducer and activator of the transcription 3 (STAT3) pathway, which exerts neuroprotective effects by releasing neurotrophic factors (Colonna and Brioschi, 2020; Giovannoni and Quintana, 2020). These processes typically stop after immune stimulation is eliminated.

However, it may be that the dynamic balance is disrupted due to the chronic inflammatory challenges or the presence of microglia dysfunction in the aging brain, which leads to the continued activation of microglia, mediating astrocyte toxicity and exacerbating inflammation response (Norden and Godbout, 2013; Spittau, 2017). Therefore, it is crucial to understand the role of neuroinflammation in the development of MCI, and as a target for therapeutic interventions.

Research on neuroinflammation-induced MCI has expanded rapidly in recent years, but the research hotspots and emerging research directions are challenging to identify. Therefore, we combed the articles in the field of neuroinflammation-induced MCI in recent 11 years, tried to establish a visual model for the study of neuroinflammatory MCI and analyze the current research hotspots, explain the evolution path of the subject in this field, and predict the future research direction. Findings from this study will not only provide a holistic picture and valuable insights on neuroinflammation-induced MCI for authors and researchers, but also a useful reference for future studies in managing neuroinflammation and treating MCI.

## Data sources and analysis methods

### Data sources

**FIGURE 1:**
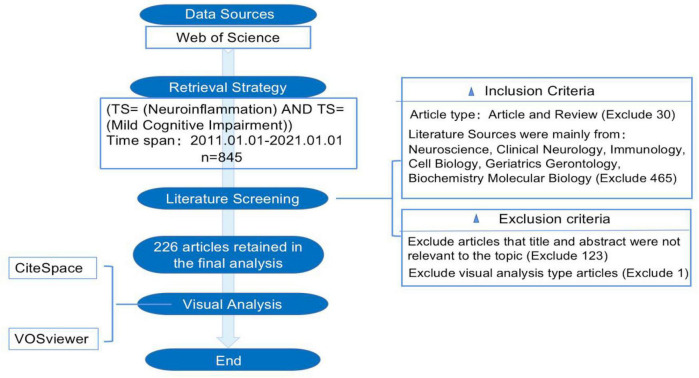
Flow chart for inclusion in literature screening.

In the core database of Web of Science (WoS), 845 relevant articles published between 2011 and 2021 were initially identified using the keywords “mild cognitive impairment” and “neuroinflammation.” The visualization analysis revealed that the literature related to the search terms was mainly focused in the fields of Neuroscience, Clinical Neurology, Immunology, Cell Biology, Geriatrics Gerontology, and Biochemistry Molecular Biology. The literature types were limited to experimental and review papers. Irrelevant literature, including visualization analysis and meta-analyses, was excluded by browsing the literature title and abstract. Finally, 226 articles meeting the inclusion criteria were selected and extracted from the WoS database. Figure 1 shows the flow chart of the literature screening.

In addition, the function of Create Citation Report in the WoS database was used for citation analysis. The sum of the number of citations and the average number of citations per item were recorded. These records, including the titles, abstracts, and cited references, were exported to CiteSpace and VOSviewer for subsequent analysis. The impact factors (IFs) of the sources were obtained from Journal Citation Reports.

### Analysis methods

CiteSpace 5. 7. R1 was developed by Dr. Chaomei Chen of Drexel University as information visualization software to identify major scientific institutions and authors, subject hotspots, and research frontiers in the scientific literature (Chen, 2004). The text data format was exported from WoS and pre-processed, then they were imported into CiteSpace for visualization and analysis. In Citespace’s visualizations map, the outermost purple ring represents the centrality of each node. The thicker the purple ring, the higher the centrality. The red ring represents a burst in a certain year. The color of the node represents a single time slice. The size of nodes and lines, respectively, represent the number of projects and the intensity of cooperation and co-occurrence.

VOSviewer 1.6.15 is free JAVA-based software developed by van Eck and Waltman of the Centre for Scientific and Technological Research at Leiden University, the Netherlands, in 2009 (van Eck and Waltman, 2010). The tab data format was exported from WoS and preprocessed, and then the data was imported into VOSviewer for visualization and analysis. In VOSviewer’s visualizations map, the size of nodes and lines represent the number of projects and the intensity of cooperation and co-occurrence, respectively. The color indicates individual time slices or the number of clusters. This study analyzed the collaborative relationships among authors, journals, the current development status, and the clustering analysis of keywords.

## Results

### Annual growth trends and basic information on publications

**FIGURE 2:**
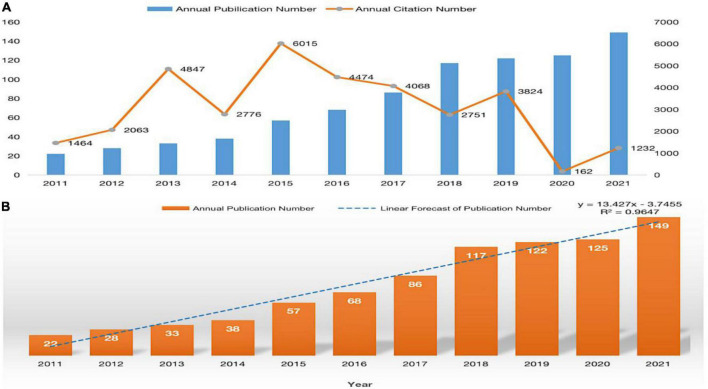
**(A)** The distribution of annual publications and citations on neuroinflammation-induced MCI, **(B)** publication forecast chart.

A total of 845 publications were identified in the WoS core database. Figure 2A shows the annual output of publications on neuroinflammation-induced MCI since 2011. The number of publications in this field has been increasing each year, and the number of publications in the past 3 years accounts for almost 50 percent of the total publications. In addition, the annual citations shows an uneven distribution. In 2015, although the number of publications was only 57, the number of citations was 6,015, the largest contributor was an article with 1,092 citations from the journal nature of Neuroscience, which belongs to medical region I and has an IF of approximately 33. Finally, the growth trend model (*R*^2^ = 0.9647) in Figure 2B predicts that approximately 156 articles on neuroinflammation-induced MCI will be published in 2022, indicating that this field continues to attract research interest from scholars and is one of the research hotspots in neurodegenerative diseases.

### Analysis of country cooperation and volume of articles issued

**FIGURE 3:**
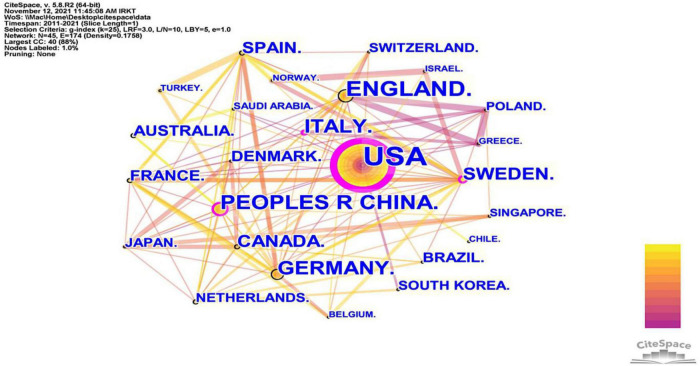
The cooperation network visualizations map of countries and regions created using CiteSpace. Node and line size represent the number of publications from a country and the cooperative relationship in the country, respectively. Color represents a single time slice. The diameter of the purple outer ring represents the size of the centrality.

Many countries have contributed to the field of neuroinflammation-induced MCI. We conducted a collaborative country network knowledge mapping study using CiteSpace for articles published from 2011 to 2021. As shown in Figure 3, only a few countries worked closely together, and most countries just stepped into this field of research. Figure 3 and Table 1 show that the United States was the leader in the number of publications, centrality, and citations. The US was followed by the United Kingdom, which ranked second in terms of the number of publications and average citations. China ranked third for the number of publications and second for centrality, and Australia was ranked last for the number of publications but had the highest average number of citations.

**TABLE 1:** Publication number, centrality, and citations by country and region.

Rank	Country	Frequency	Centrality	Total citations	Average citations
1	All countries	281	N/A	34,669	123.38
2	United States	92	0.54	7,281	79.14
3	England	34	0.09	3,936	115.76
4	China	31	0.17	4,821	155.52
5	Italy	27	0.16	1,103	40.85
6	Germany	25	0.08	3,767	150.68
7	Sweden	18	0.14	2,829	157.17
8	Canada	18	0.07	2,725	151.39
9	Spain	14	0.06	2,617	186.93
10	France	12	0.04	2,763	230.25
11	Australia	10	0.00	2,827	282.70

### Analysis of author and co-author collaboration and volume of publications

**FIGURE 4:**
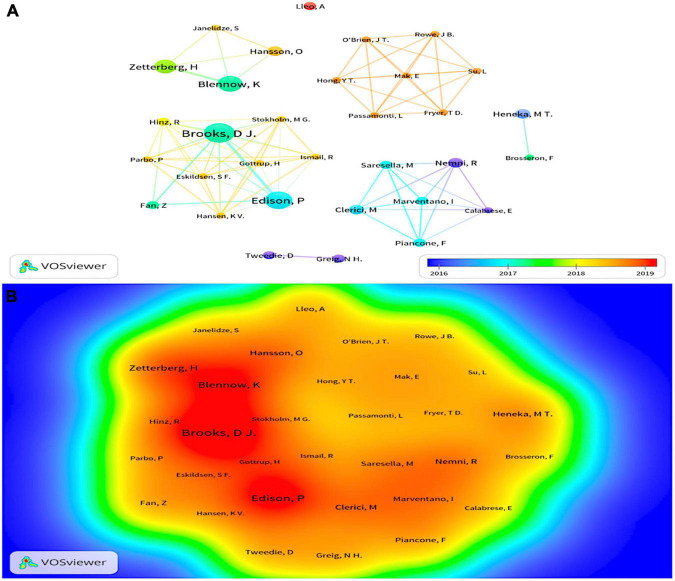
**(A)** The cluster visualization map of cited authors created using VOSviewer from 2016 to 2019, **(B)** the density visualization map of cited authors created using VOSviewer. Node size and color represents total number of citations and a single time slice, respectively. The darker the color, the more publications.

Since 2011, 1,483 authors have participated in research in the field of neuroinflammation-induced MCI. In Figure 4A, VOSviewer’s author collaboration mapping revealed seven collaborations between cited authors who were not closely related, and the publications were mainly concentrated between 2016 and 2019. Table 2 shows the chief five authors and co-authors. The top author was Brooks D.J. from Denmark (h index = 130) with 10 publications, the color around this name in Figure 4B is the darkest, indicating that this author had the highest number of publications. The second- and third-ranked authors were Edison P. and Blennow K., respectively, both with eight publications. The top co-cited authors were Heneka M.T., Petersen R.C., and Jack C.R. Heneka M.T., they contributed the most in this field, and the representative work “Neuroinflammation in Alzheimer’s disease” was the most frequently cited paper in the search results, this paper was published in 2015 in a top journal, Lancet Neurology.

**TABLE 2:** Number of publications of the top five cited authors and co-authors.

Author	Region	Frequency	Citations	Co-author	Region	Frequency	Co-citations
Brooks DJ	Denmark	10	2,914	Heneka M.T.	Germany	69	4,946
Edison P	England	8	590	Petersen R.C.	United States	49	3,174
Blennow K	Sweden	8	393	Jack C.R.	United States	44	1,654
Zetterberg H	Sweden	7	395	Braak H.	Germany	38	1,452
Heneka MT	Germany	6	2,777	McKhann G.M.	United States	36	1,209

### Analysis of journal collaboration and volume of publications

**FIGURE 5:**
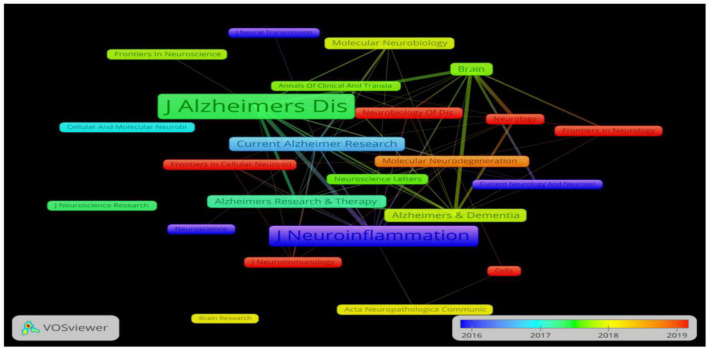
The cooperation network visualization map of journals created using VOSviewer for articles published from 2016 to 2019. Node size and color represents total number of citations and a single time slice, respectively.

A total of 57 journals related to the field of neuroinflammation-induced MCI were included in this data analysis. Figure 5 shows the journal collaboration mapping created using VOSviewer, which shows that the *Journal of Neuroinflammation* and *Journal of Alzheimer’s Disease* had the highest volume of relevant published literature. The top five journals in terms of the number of publications are listed in Table 3. The top journal was the British *Journal of Neuroinflammation* (IF = 8.322), with 31 publications related to neuroinflammation and MCI focusing on components related to innate immune responses in the central nervous system, including microglia, astrocytes, and various cytokines and chemokines. These immune responses, which are central to the pathogenesis of various neurological disorders, are an area of notable research advances. The *Journal of Neuroinflammation* was followed by the British journal *Brain* (IF = 13.501) and the Dutch *Journal of Alzheimer’s Disease* (IF = 4.472).

**TABLE 3:** Top five journals in terms of the number of publications.

Rank	Journal	Frequency	Citations	IF (2020)	Region
1	*J Neuroinflammation*	31	1,086	8.322	England
2	*Brain*	9	731	13.501	England
3	*J Alzheimer’s Dis*	41	687	4.472	The Netherlands
4	*Alzheimer’s and Dementia*	11	630	21.566	United States
5	*Molecular Neurobiology*	8	463	5.59	United States

### Analysis of institutions’ collaboration and volume of publications

**FIGURE 6:**
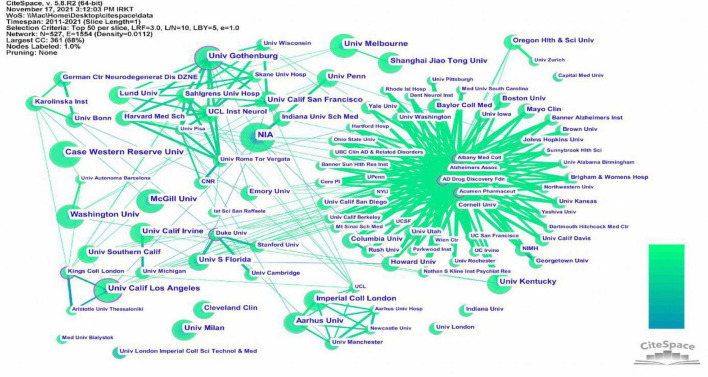
The cooperation network visualizations map of institutions created using CiteSpace. Node and line size represent the number of publications from an institution and the cooperative relationship in the institution, respectively. Color represents a single time slice. The diameter of the purple outer ring represents the size of the centrality.

This study included publications from 527 institutions, and the inter-institutional linkages were visually analyzed using CiteSpace software. The information on communication and collaboration between different institutions is presented in Figure 6. The results show that Albany Medical College, AD Drug Discovery Foundation, and Acumen Pharmaceuticals have the most clearly defined purple outer circles. The affiliated institutions are distributed around them, indicating that these three institutions had higher centrality than other institutions. To understand the details of the published articles of these institutions, these institutions were ranked in terms of the number of published articles. And the top five institutions were selected and listed in Table 4. This table shows that the NIA was ranked first, with ten published articles and a centrality of 0.09. Three of the top five institutions are in the United States, suggesting that many institutions in the United States are engaged in research on neuroinflammation and MCI.

**TABLE 4:** Institutions’ publication number, country, and first publication year.

Institution	Counts	Centrality	Region	First year
National institute on aging	10	0.09	United States	2011
Case western reserve university	9	0.00	United States	2015
University of California Los Angeles	8	0.11	United States	2012
University of Gothenburg	8	0.11	Sweden	2014
University of Melbourne	8	0.06	Australia	2014
				

### Analysis of changes in research themes

Reference co-citation analysis is essential for analyzing trends and identifying research opportunities in a given field. For this purpose, we used CiteSpace to analyze co-cited references. In Table 5, the top five references selected include three reviews and two human experiments, and all of these articles are related to Alzheimer’s disease and neuroinflammation.

**TABLE 5:** Top five co-cited references.

Publication	Citation	Author (Year)	Co-citations
NIA-AA research framework: toward a biological definition of Alzheimer’s disease	16	Jack et al., 2018	3,285
Neuroinflammation in Alzheimer’s disease	32	Heneka et al., 2015	3,196
Immune attack: the role of inflammation in Alzheimer disease	15	Heppner et al., 2015	1,530
Early and protective microglial activation in Alzheimer’s disease: a prospective study using F-18-DPA-714 PET imaging	16	Hamelin et al., 2016	273
Influence of microglial activation on neuronal function in Alzheimer’s and Parkinson’s disease dementia	13	Fan et al., 2015	140

**FIGURE 7:**
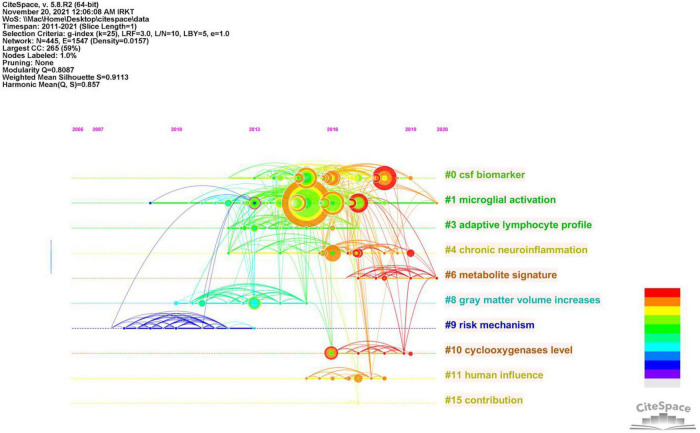
Timeline of co-citated document clusters from 2011 to 2021. Node size and color represents total number of citations and a single time slice, respectively. Lines of different colors show that the two articles were co-cited in an article.

To better understand the current research themes and predict future directions in the field of neuroinflammation-induced MCI, we created a timeline diagram based on CiteSpace for the period 2011–2021 (Figure 7). This diagram is a knowledge network graph with 445 nodes and 1,547 links. A total of 10 reference clusters are listed in the figure, and their ranking is determined by the amount of literature contained. The clusters are described in Table 6, each group was assigned a silhouette value ranging from –1 to 1, and a value greater than 0.7 indicated that the cluster had a good range of identification.

**TABLE 6:** Research frontier identification.

Cluster	Size	Silhouette	Year	Description
0	55	0.857	2016	Altered bile acid profile in mild cognitive impairment and Alzheimer’s disease: relationship to neuroimaging and cerebrospinal fluid biomarkers.
1	49	0.827	2015	*In vivo* imaging of glial activation in Alzheimer’s disease.
3	32	0.925	2014	Peripheral immune system in aging and Alzheimer’s disease.
4	27	0.950	2016	Amyloid-beta, tau, and the cholinergic system in Alzheimer’s disease: seeking direction in a tangle of clues.
6	25	0.926	2018	Dysregulation of systemic immunity in aging and dementia.
8	20	0.987	2012	Cerebrospinal fluid sTREM2 levels are associated with gray matter volume increases and reduced diffusivity in early Alzheimer’s disease.
9	20	0.991	2010	Cerebrospinal fluid sTREM2 levels are associated with gray matter volume increases and reduced diffusivity in early Alzheimer’s disease.
10	17	0.965	2017	Involvement and relationship of bacterial lipopolysaccharide and cyclooxygenase levels in patients with Alzheimer’s disease and mild cognitive impairment.
11	16	0.969	2017	IL-1 beta, IL-6, IL-10, and TNF-alpha single nucleotide polymorphisms in human influence the susceptibility to Alzheimer’s disease pathology.
15	4	1	2017	HIV-1 infection alters energy metabolism in the brain and contributions to HIV-associated neurocognitive disorders.
				

**FIGURE 8:**
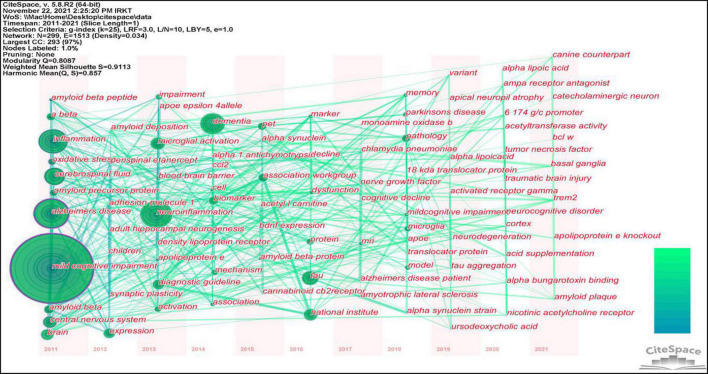
The co-citation network visualizations map of keywords created using VOSviewer. Node size and color represents the number of keywords and cluster.

Figure 8 and Table 7 show that the main research topics of MCI and neuroinflammation were related to studying various cell-intrinsic molecular mechanisms and metabolites. As shown in Figure 8, the publications in Cluster 0 were associated with cerebrospinal fluid biomarkers, and 55 references were included in this cluster. Biologically relevant cerebrospinal fluid markers have been highly researched in combination with time. Studies have found that elevated levels of inflammation-related features are detected in the cerebrospinal fluid and serum in patients with MCI and AD patients [e.g., YKL-40, interleukin (IL)-8, TREM2, and monocyte chemotactic protein-1 (MCP-1)] (Bowman et al., 2018; Nordengen et al., 2019).

**TABLE 7:** Keywords with the highest co-occurrence frequency.

Rank	Frequency	Keyword	Centrality
1	92	Mild cognitive impairment	0.19
2	44	Alzheimer’s disease	0.22
3	38	Neuroinflammation	0.08
4	35	Inflammation	0.09
5	29	Dementia	0.04
6	28	Cerebrospinal fluid	0.22
7	24	Mouse model	0.14
8	22	Tau	0.06
9	21	Central nervous system	0.18
10	18	Microglial activation	0.04

The focus of publications in Cluster 1 was microglia activation (the primary driver of neuroinflammation-induced MCI), which was the most heavily studied topic from 2013 to 2018 and became a hot research topic again in 2020.

The research in Cluster 3 focused on neuroinflammation and adaptive immune cells. Although there have been few studies on adaptive immune cells in recent years, their role cannot be ignored. One study identified CD4 T cells in the brains of healthy mice, and these cells promoted the development and maturation of microglia (Pasciuto et al., 2020). In mouse models, therapeutic depletion of B cells at disease onset delayed AD progression, suggesting that treatment targeting B cells may benefit patients with AD (Kim et al., 2021). The close functional link between the immune and the central nervous systems has increased recognition.

Publications in Cluster 4 focused on Neuroinflammation, which plays a vital role in neurodegenerative diseases. In the earliest stages, the initiation of glial cells, the release of pro-inflammatory factors, and the vicious cycle of neuronal damage cause irreversible damage; therefore, identifying the neural mechanisms of neuroinflammation-induced degenerative diseases is crucial for solving the problem (Needham et al., 2022).

Publications in Cluster 10 studied elevated levels of cyclic oxygenase (COX-1/2). COX-1/2 is an essential modulator of brain plasticity, but its overexpression in neurons can increase the progression of brain injury and mediate the expression of other inflammatory factors, accelerating neuronal death (Andreadou et al., 2021).

### Analysis of keywords

#### Research hotspot analysis

Table 6 shows that the keywords with the highest frequency were mild cognitive impairment, Alzheimer’s disease, neuroinflammation, inflammation, cerebrospinal fluid, mouse model, tau, central nervous system, and microglial activation.

**FIGURE 9:**
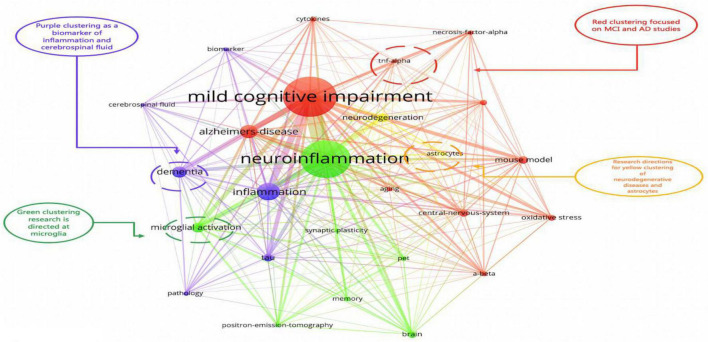
The co-citation timeline zone of keywords created using CiteSpace. The size of the node reflects the co-occurrence frequencies, and the link indicates the co-occurrence relationship. Nodes and lines change over time from 2012 to 2020.

This study identified 1,466 keywords, and the keyword clustering was visualized using VOSviewer (Figure 9). It was noted that many of the keywords shown in Figure 8 appeared in highly co-cited references. The keywords were categorized into red, green, purple, and yellow clusters, representing four research directions. The main keywords in the red cluster are MCI, AD, A-beta, oxidative stress, and central nervous system. The main keywords in the purple cluster are tau, biomarkers, dementia, and cerebrospinal fluid, the main keywords in the green cluster are neuroinflammation, microglial activation, brain, memory, and PET, and the main keywords of the yellow cluster mainly are astrocytes and neurodegeneration.

One study showed that two-thirds of patients with AD were converted from MCI (Petersen, 2004). Aβ plaque deposition and neuronal dysfunction occur in the brain of patients with MCI, and Aβ plaque deposition is also a prominent pathological feature of AD. Microglia and astrocytes are the primary mediators of neuroinflammation. Under physiological conditions, microglia monitor the CNS microenvironment and its processes to sense injury signals; they are the first cells to sense inflammatory stimuli and, along with astrocytes, maintain CNS homeostasis (Leng and Edison, 2021). The function of both cell types is to clear Aβ plaques to restore neuronal health. In the aging brain, this function is probably compromised because of the saturation of microglia phagocytosis or the dysfunction of microglia, which limits Aβ clearance. Moreover, microglia are in a constant state of activation, and the release of large amounts of pro-inflammatory cytokines triggers neuroinflammation, leading to neuronal dysfunction and death. In addition, saturated cells recruit more microglia and peripheral macrophages to accumulate around Aβ plaques, disrupting the balance of pro-inflammatory and anti-inflammatory factors in the brain and triggering chronic inflammation (Kinney et al., 2018). In an *in vitro* study, microglia responded differently to different Aβ species, phagocytosing Aβ fibers rather than Aβ oligomers, which preferentially induced inflammatory cytokine production and inhibited the phagocytosis of Aβ fibers (Pan et al., 2011). The stimulation of small oligomers of Aβ is much greater than that of large oligomers of Aβ, inducing an activation response in microglia and producing more significant neurotoxicity (Del Bo et al., 1995; Akiyama et al., 2000). Tau is another pathological feature of AD. However, the correlation between tau deposition and microglial activation in patients with MCI is weak, and this correlation becomes more vital only in the development of the AD stage (Dani et al., 2018). *In vitro* studies have revealed that tau oligomers and fibers provide sufficient stimulation to induce microglia morphological changes and IL expression. Further *in vivo* studies using the tau mouse model confirmed the presence of tau-induced microglia activation, marked by the upregulation of DAM-related genes (disease-associated microglia) (Wes et al., 2014). In addition, most neuroimaging studies have been performed with PET and radioligands, which target transporter proteins (TSPO) to provide additional information on different glial cell phenotypes (Zimmer et al., 2014).

The interaction between microglia and astrocytes also significantly affects neuroinflammation. Microglia and astrocytes are part of the neurovascular unit (NVU) and are activated by various brain injuries (Liu et al., 2020). Microglia are more sensitive to damage, they are first activated into the M1 phenotype by pathogen-associated molecular pattern molecules (PAMPs) and damage-associated molecular pattern molecules (DAMPs) and promote the secretion of inflammatory factors (e.g., TNF-a and IL-1) to trigger reactive astrocytes (A1). With damage limitation and NVU remodeling, the local environment is altered, and microglia shift to the M2 phenotype (Liddelow et al., 2017). At the same time, local environmental factors may facilitate the conversion of astrocytes to A2, promoting neuronal survival and repair. It has been reported that a single astrocyte can supervise more than 100,000 synapses (Bushong et al., 2002; Wang et al., 2006; Halassa et al., 2007; Allen et al., 2012). Astrocytes extend terminal protrusions at approximately 99% to cover the surface of brain vessels to regulate cerebral blood flow (CBF) or the BBB (Filosa et al., 2016). Moreover, astrocytes can organize into syncytial structures of up to 100 units through gap junctions to facilitate long-distance signaling (Giaume, 2010; Charvériat et al., 2017). As mentioned above, when injury and neuronal death increase, M1 microglia trigger more reactive astrocytes (A1) *via* inflammatory signaling inflammation, thereby amplifying cascading neuroinflammation (Liu et al., 2020).

#### Research frontier identification

Figure 8 plots the keywords throughout 2011–2021, showing the changes in the core research areas in the past decade. The position of the keyword circles represents the year in which the keyword first appeared, and the lines between circles represent the connections between each keyword. The circle for the keyword “mild cognitive impairment” is the largest, indicating the most common keyword used. TREM2 and Bcl-w were among the emerging keywords in 2021, and recent studies have suggested that TREM2 is the most potent risk factor for AD. In one study, TREM2 was also shown to suppress inflammatory responses in mouse microglia by inhibiting PI3K/NF-κB signaling (Li et al., 2019). Under the relevant conditions of AD, TREM2 interacted with lipoproteins, anionic lipids, and Aβ, contributing to the microglial promotion of amyloid plaque deposition and phagocytosis of cellular tissue debris (Xue and Du, 2021). In another study, TREM2 deficiency in the presence of Aβ pathology promoted tau protein accumulation, proliferation, and brain atrophy, which facilitated microglia survival and conversion to DAM and exacerbated the disease process (Haass, 2021). These findings can be attributed to differences between animal models or experimental disease stages, and the conflicting results emphasize the importance of careful evaluation. The mechanism of action of TREM2–ligand interaction remains unclear and requires further studies.

## Discussion

From a bibliometric perspective, the increasing number of studies on neuroinflammation-induced MCI indicates a surge of interest in this research area. In the graph of the predicted number of publications in the literature, it was found that the number of papers related to neuroinflammation-induced MCI increased over time. However, there was no significant increase in magazines between 2019 and 2020, this may have been associated with COVID-19, which affected global academic communication and slowed most scientific research considerably. The country analysis showed that the research efforts in this field are uneven among countries; developed countries, led by the United States, have produced the most publications and have the highest centrality, which may be related to the high level of economic development of the country and the importance it places on this field. Regarding journals and institutions, most of those in the top positions are from highly developed Western countries, such as the United States, the United Kingdom, and the Netherlands, which may be related to national policies and financial support.

Publications with co-cited authors such as Brooks D.J., Edison P., Blennow K., Zetterberg H., and Heneka M.T. have significantly influenced current research trends and the current understanding of neuroinflammation-induced MCI. Heneka et al. (2015) focused on neural inflammation in AD, they elucidated the role of microglia and astrocytes in neuroinflammation and provided an outlook on future directions in the field. Edison (2021) focused on the potential mechanisms of glial cell activation, local and systemic inflammation on disease progression, and target translocator protein (TOSP) to assess the relationship between Aβ deposition and neuroinflammation. Pascoal et al. evaluated the association between microglia and tau and found that microglial activation was associated with soluble TREM2 in the cerebrospinal fluid (Pascoal et al., 2021). Teunissen et al. provided information about the potential of AD progression and detection of treatment effects, based mainly on studies of neurodegeneration markers in the blood (Teunissen et al., 2022). Most of these researchers have made significant contributions in the field of neuroinflammation-induced MCI. In the future, researchers should enhance their collaboration efforts to maximize advances in the related field.

The publications covered in the analysis of research themes are the most influential in this field and they represent, to some extent, a change in research trends. As these themes have changed in recent years, the effects of metabolites on the brain and microglial activation have begun to draw attention. The use of metabolomics to measure the biochemical products produced by cellular processes can measure alterations in the biochemical pathways of disease and explore disease markers (Varma et al., 2018). The concentrations of the critical markers Aβ1-42, T-tau, P-tau181, neurofilament light chain (NfL), and glial fibrillary acidic protein (GFAP) in the cerebrospinal fluid have diagnostic value in diagnosing AD (Bjerke and Engelborghs, 2018). A recent study found that plasma concentrations of the phospholipid metabolite 2-aminoethyl dihydrogen phosphate normalized to taurine concentration could better identify patients with early AD (Banack et al., 2022).

Through a keyword analysis of neuroinflammation-induced MCI, it was possible to identify that neurotoxicity is mainly mediated by microglia and astrocytes. The interaction between them may be an effective and precise therapeutic target in the future (Liu et al., 2020). This represents a new research direction for MCI. In the early stages of MCI, activated microglia are initially protective, but as the disease progresses, the chronic activation of microglia results in a pro-inflammatory phenotype. This leads to damage to neuronal networks, which may be due to acquired or genetic factors, impairing microglia’s protective function. For example, the APOE-ε4 allele is the most potent genetic risk factor for sporadic AD. The upregulation of the APOE-ε4 allele has been found in microglia in diseased brains, and APOE-ε4 increases microglia activation responses and converts microglia to a pro-inflammatory phenotype (Grubman et al., 2019).

In addition, the phenotypic changes in microglia are associated with TREM2, whose activation induces an APOE-ε4-dependent pathway in microglia, exacerbating the loss of homeostatic function and the up-regulation of pro-inflammatory molecules in microglia (Krasemann et al., 2017). In recent studies on microglial phenotypes, CD33 has been identified as a sialic acid-binding immunoglobulin-type lectin receptor on microglia. The long CD33M subtype inhibits microglial phagocytosis, migration, and proliferation, whereas the short subtype increases microglia proliferation; furthermore, the CD33 receptor on microglia is dependent on the presence of TREM2 (Griciuc et al., 2019). Because TREM2 is an essential upstream mediator of microglia activation and phenotypic changes, it is also an attractive target for drug regulation (Keren-Shaul et al., 2017; Krasemann et al., 2017). Therefore, in the future study, exploring the inner link between phenotypic changes in microglia and neuroinflammation may provide a potential target for treating MCI and preventing AD.

Recent studies have shown that women over the age of 65 are more likely to develop AD than men in the same age group, and that hippocampal atrophy is more severe in women than in men. Genetic dysregulation of microglia was also found in animal models, particularly in the temporal cortex and parahippocampal gyrus. This dysregulation was more pronounced in females, and microglia activation, amyloid deposition, and behavioral changes occurred earlier in female mice. However, the clinical evidence is limited because the female population was under-sampled, leading to unbalanced samples and gender bias. Future studies should focus on gender differences to elucidate the specific disease mechanisms (Lynch, 2022). Evidence suggests that gut microbes can influence neuroinflammation in the brain *via* the microbiota-gut-brain axis. Under pathological conditions, the abundance of anti-inflammatory flora decreases but the pro-inflammatory flora increases, which cause dyshomeostasis of intestinal flora. This results in a more robust inflammatory response and the subsequent infiltration of inflammatory factors from the intestinal epithelium into the brain’s circulation in a vagal manner leading to neuroinflammation (Bonaz and Bernstein, 2013; Bairamian et al., 2022). In one study, analyzed of the gut microbial metabolomic analysis of patients with MCI and AD revealed alterations in the fecal microbiological profile of patients with AD, mainly focusing on indole-3-pyruvic acid (IPyA), which may be a mediator of intestinal ecological dysregulation and could be involved in the development of AD (Wu et al., 2021). Therefore, in future studies, exploring the inner link between IPyA and neuroinflammation may provide a potential target for treating MCI and preventing AD.

In addition, inflammatory factors can disrupt the BBB and thus allow leukocytes and other cells to infiltrate the brain, which will exacerbate neuroinflammation and act directly on microglia. The ability of microglia to monitor pathogens and cellular debris is thus disrupted, along with their ability to phagocytose Aβ (Bonaz and Bernstein, 2013; Bairamian et al., 2022). Therefore, targeting the microbiota rather than immune function appears feasible for preventing and controlling MCI.

In terms of current advances in treating MCI by targeting neuroinflammatory molecules, a clinical study demonstrated that subcutaneous injections of IFNβ1a could modulate neuroinflammation-induced MCI showed that the treatment tended to slow disease progression (Grimaldi et al., 2014). In addition, several other anti-inflammatory factors, including IL-2, IL-4, and IL-33, have the potential to attenuate microglial activation in the clinical setting. Tanshinone IIA reduced microglia and astrocyte activation and prevented neuronal synapse loss in APP/PS1 mice by inhibiting the RAGE/NF-κB signaling pathway *in vivo* and vitro (Ding et al., 2020). In APP/PS1 transgenic mice, the PPARγ agonists (pioglitazone and rosiglitazone) shifted microglia from the pro-inflammatory phenotype to the phagocytic phenotype and promoted amyloid clearance (Escribano et al., 2010; Yamanaka et al., 2012). In addition, exercise therapy and a healthy lifestyle were recently identified as non-pharmacological treatments that can mitigate chronic cognitive disorders, including MCI (Zotcheva et al., 2021).

## Conclusion

Findings from this study show that research on neuroinflammation-induced MCI is burgeoning, and the close collaboration with different nations and institutions need to be further strengthened. Current research hotspots are focused on the effects of various metabolites on microglia activation. Future studies should focus on how to reduce smation and treat MCI *via* inhibiting the pro-inflammatory phenotype and enhancing the anti-inflammatory phenotype of microglia and astrocytes.

## Author contributions

MC and LZ conceived the study. YZ, SC, and ZW collected the data. YZ wrote the article. MC revised the article. All authors contributed to the article and approved the submitted version.
